# A Multi-Parameter, High-Content, High-Throughput Screening Platform to Identify Natural Compounds that Modulate *Insulin* and *Pdx1* Expression

**DOI:** 10.1371/journal.pone.0012958

**Published:** 2010-09-23

**Authors:** Jessica A. Hill, Marta Szabat, Corinne A. Hoesli, Blair K. Gage, Yu Hsuan C. Yang, David E. Williams, Michael J. Riedel, Dan S. Luciani, Tatyana B. Kalynyak, Kevin Tsai, Ziliang Ao, Raymond J. Andersen, Garth L. Warnock, James M. Piret, Timothy J. Kieffer, James D. Johnson

**Affiliations:** 1 Department of Cellular and Physiological Sciences, University of British Columbia, Vancouver, British Columbia, Canada; 2 Department of Surgery, University of British Columbia, Vancouver, British Columbia, Canada; 3 Department of Chemical and Biological Engineering, University of British Columbia, Vancouver, British Columbia, Canada; 4 Departments of Chemistry and Earth and Ocean Sciences, University of British Columbia, Vancouver, British Columbia, Canada; Louisiana State University, United States of America

## Abstract

Diabetes is a devastating disease that is ultimately caused by the malfunction or loss of insulin-producing pancreatic beta-cells. Drugs capable of inducing the development of new beta-cells or improving the function or survival of existing beta-cells could conceivably cure this disease. We report a novel high-throughput screening platform that exploits multi-parameter high-content analysis to determine the effect of compounds on beta-cell survival, as well as the promoter activity of two key beta-cell genes, *insulin* and *pdx1*. Dispersed human pancreatic islets and MIN6 beta-cells were infected with a dual reporter lentivirus containing both eGFP driven by the *insulin* promoter and mRFP driven by the *pdx1* promoter. B-score statistical transformation was used to correct systemic row and column biases. Using this approach and 5 replicate screens, we identified 7 extracts that reproducibly changed *insulin* and/or *pdx1* promoter activity from a library of 1319 marine invertebrate extracts. The ability of compounds purified from these extracts to significantly modulate *insulin* mRNA levels was confirmed with real-time PCR. Insulin secretion was analyzed by RIA. Follow-up studies focused on two lead compounds, one that stimulates insulin gene expression and one that inhibits insulin gene expression. Thus, we demonstrate that multi-parameter, high-content screening can identify novel regulators of beta-cell gene expression, such as bivittoside D. This work represents an important step towards the development of drugs to increase insulin expression in diabetes and during *in vitro* differentiation of beta-cell replacements.

## Introduction

The survival and function of the pancreatic beta-cell is critical in the pathogenesis of all forms of diabetes mellitus [1], [2], [3], [4], [5]. Increased beta-cell apoptosis and decreased beta-cell function are also major complicating factors in clinical islet transplantation [6], [7], [8], [9], [10]. Thus, efforts to generate new beta-cells or increase the function and survival of beta-cells are critical. To date, significant progress has been made in understanding the control of beta-cell function and fate by focusing on specific candidate genes and pathways [11], [12], [13], [14], [15], [16]. Similarly, high throughput screens for novel compounds affecting beta cell function have relied on single reporters typically transfected in non beta-cell lines [17], [18]. High throughput screening has recently been employed to direct embryonic stem cells towards a beta-cell lineage [19], to increase insulin expression in an alpha-cell line [20] and to increase beta-cell survival [21]. As a step towards the goal of increasing beta-cell function, proliferation or survival, we designed a multi-parameter, high content screening platform that enabled us to screen a library containing 1319 unique natural marine invertebrate extracts. We simultaneously examined four parameters for each compound, including *insulin* promoter activity, *pdx1* promoter activity, nuclear morphology, and cell number. Insulin was chosen as a primary parameter because it defines the fitness and maturity of pancreatic beta-cells [22]. *Pdx1* was chosen because it is widely considered to be a master beta-cell transcription factor, controlling both the survival and function of this cell type [14], [23], [24], [25]. Nuclear morphology and cell number were convenient indices of apoptosis and cell viability [14].

Here, we outline our implementation of image analysis strategies and statistical tools to distill large high-content data sets. Specifically, we use a B-score transformation to effectively mitigate the systematic row and column artifacts that are commonly observed during high-throughput screens of live cells. Our results demonstrate the feasibility of multi-parameter, high-content screening in beta cell lines and primary human islets. We identify novel compounds that can significantly increase or decrease *insulin* gene expression.

## Methods

### Lentiviral fluorescent reporters, cell culture and marine extract library

The dual reporter and control lentiviral vectors were generated and validated as previously described [22], [26]. Primary human islets were provided by the Centre for Human Islet Transplantation and Beta-cell Regeneration. All procedures related to the isolation and research use of human islets were approved by the local institutional review board in accordance with international and national guidelines. Islets were cultured overnight at 37°C and 5% CO_2_ in RPMI1640 medium supplemented with 5 mM glucose (Sigma), 100 units/mL penicillin, 100 µg/mL streptomycin (Invitrogen, Burlington, ON), and 10% fetal bovine serum (FBS: Invitrogen) as described previously [10], [27], [28]. The MIN6 insulinoma cell line [29], obtained under MTA from Jun-Ichi Miyazaki (Osaka University, Japan), was cultured (passages 25–39) in Dulbecco's modified Eagle's medium containing 25 mM Glucose, 100 units/mL penicillin, 100 µg/mL streptomycin and 10% FBS [12], [22], [27], [30]. MIN6 cells and dispersed human islet cells were infected as previously described [22], [26]. These labeled cells were used to screen a library of marine invertebrate extracts that has been detailed elsewhere [31]. Additional information is available in the Supplement and Supplementary Figures S1 and S2.

### Image acquisition, object identification, B-score data transformation

Images were acquired by automated fluorescence microscopy using the Cellomics ArrayScan V^TI^ (Thermo Fisher). Object (cell) identification was performed using the Cellomics Target Activation algorithm. For each cell/object identified, average and total intensity and intensity variance measurements were taken. Additional details are presented in the Supplement. Data were transformed to mitigate systematic row and column effects using the B-score method [32], [33], [34] as described in detail in the Supplement. All B scores were calculated using the package *cellHTS* [33] for R or, subsequently, using code we have designed for R written by J. Hill.

### Compound Purification

A specimen of the freeze-dried sea cucumber (161 g) collected in Pohnpei was extracted exhaustively with MeOH (3×100 mL) at room temperature. After evaporation the crude extract was partitioned between H_2_O (1×15 mL) and EtOAc (4×5 mL) followed by n-BuOH (4×5 mL). The combined dried active n-BuOH extract was then fractionated by Sephadex LH20 column chromatography to give an active fraction consisting of bivittoside D. We confirmed that this active fraction of bivittoside D was 95% pure, with the remainder being bivittoside C and other related analogues using MALDITOF-MS ([M+Na]+ at m/z 1449.8). The structure was confirmed by analysis of the 1H and 13C NMR spectra.

### Gene expression analysis

MIN6 cells were plated at 50–70% confluency in tissue culture treated 12-well plates, (NUNC, VWR, Mississauga, ON, Canada) and treated for 18–24 hours with 2 µl/ml of serially diluted (1×10^−1^, 1×10^−2^, 1×10^−3^) crude and butanol extracts in DMEM media. Trizol-isolated total RNA was purified using the RNeasy kit, Qiagen (Mississauga, ON, Canada). DNase-treated total RNA (100ng) was converted to cDNA by qScript cDNA synthesis kit, (Quanta BioSciences, Inc., VWR, Mississauga, ON, Canada). Real-time PCR was performed on Applied Biosystems StepOnePlus™ platform. Custom designed TaqMan primers for *Insulin1*, *Insulin2* and *cyclophilin* were purchased from Integrated DNA Technologies (IDT, Coralville, IA). Relative changes in gene expression were analyzed by real-time PCR using the 2^−ΔΔCt^ method. Sequences for primers and probes are available in the Supplement.

### Insulin secretion

MIN6 cells were treated with compounds, as indicated, for 18–24 hours in 25 mM glucose containing DMEM media supplemented with 10% FBS. Media were collected and centrifuged at 5000g for 5 minutes to remove any cellular debris and insulin levels were assayed with a rat insulin radioimmunoassay kit (Linco Research, St Charles, MO, USA) [35].

### Statistics

The R environment for statistical computing (http://www.R-project.org) was used for data analysis during the screening process, unless otherwise indicated. In all cases, unless otherwise mentioned, five replicate experiments were performed and statistical tests were considered significant at an alpha level of 0.05. For follow-up studies, Graphpad Prism or Microsoft Excel were used to perform ANOVA or t-tests as appropriate. Results were considered significant if the *P* value was less than 0.05.

## Results

### Overall design of the high-content screen

**Figure 1:**
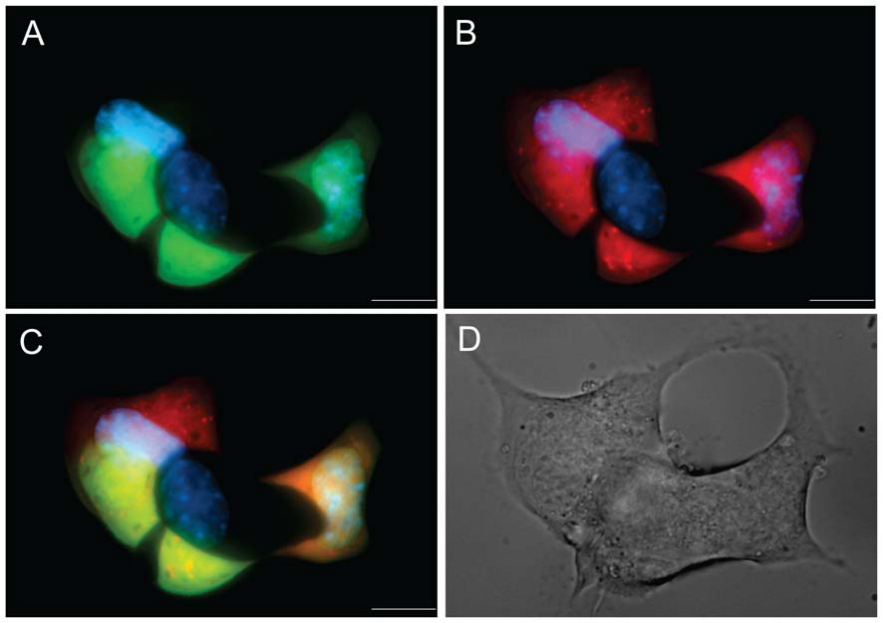
Fluorescent protein expression in living beta-cells infected with dual reporter lentivirus. (A,B) A representative group of MIN6 cells is imaged in the EGFP or RFP channels. (C) Merged image shows the heterogeneity in *insulin* and *pdx1* promoter activity between single cells. Hoechst was used to counterstain the nuclei. (D) Differential interference contrast microscopy image of the MIN6 cells from panels A–C.

We designed a multi-parameter, high-content, high-throughput screen to identify natural extracts that modulate beta-cell survival and function. The screen was designed to provide sensitive real-time analysis of the expression of two key genes, namely *insulin* and *pdx1* in live beta-cells (Fig. 1). *Pdx1* plays a positive role in *insulin* gene transcription [36], giving the screen an element of built-in redundancy. However, the requirement for *Pdx1* for *in vivo Insulin* expression is not absolute [14], [23], [37], [38], [39], [40], suggesting that each gene reporter should be evaluated independently. The chosen parameters would also likely allow the identification of compounds capable of promoting the maturation of *pdx1*-positive immature beta-cells towards the fully differentiated state of adult beta-cells [22], [26]. We also reasoned that it would be critical to examine the effect of all of crude extracts on cell viability. For this we employed two complementary approaches, a simple count of cell number and the analysis of nuclear DNA condensation (apoptosis). The replicative capacity of adult primary beta-cells is very low [41], [42], suggesting changes in cell number would likely reflect changes in survival and the rapid proliferation of non-beta-cells. In contrast, MIN6 beta-cells replicate quickly and represent a comparably homogenous cell population, suggesting that the cell number parameter would also represent a good surrogate for proliferative activity of the extracts.

### Data summarization and transformation

Our screen was designed with two key assumptions. First, we assumed that biologically relevant hits would occur only with a very small minority of all extracts tested. This meant that we could use the majority (>99%) of the extracts as negative controls. Second, we assumed that experimental variation between identical treatments would be similar to our typical experiments that examine gene expression and cell viability. Thus, we replicated our MIN6 cell screen 5 independent times. Due to low donor availability, low beta-cell purity in some batches of islets and low infection efficiency with some batches of virus, we did not have the same luxury with the human islet preparations. Only one batch of human islets met our standards for purity (>80%) and high lentiviral infection.

**Figure 2:**
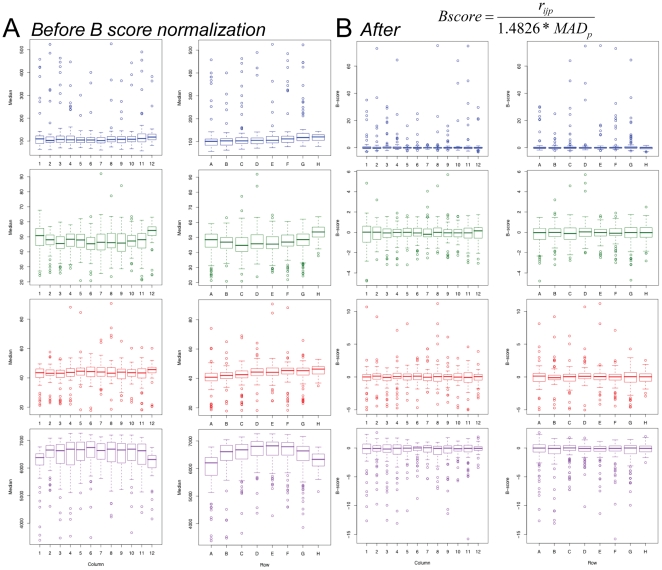
Correction of systematic row and column biases in 96-well format high-throughput data using a B score transformation. (A) Overall column and row effects on medians of multiple parameters. The overall bias is shown for measurements of nuclear intensity (Hoechst; blue box plots), *insulin* expression (green), *pdx1* expression (red) and cell number (purple). Box plots represent intraquartile range (IQR) with the dark line representing the median. Whiskers represent 1.5*IQR and outliers are any values exceeding that. (B) These systematic biases are corrected using a B score transformation. Overall column and row effects on cell measured after B-score transformation. Box plots for nuclear intensity (Hoechst; blue box plots), *insulin* (green), *pdx1* (red) and cell number (purple). Box plots represent IQR with the dark line representing the median. Whiskers represent 1.5*IQR and outliers are any values exceeding that.

Image data were summarized using a median-based approach that is described in more detail in the Supplemental Methods S1. We examined our large data set for systematic biases. Both row and column biases have been noted by others when performing high-throughput screening in the 96-well plate format [43]. Indeed, we observed consistently that the outside rows and columns had lower values in several parameters (Fig. S3). The ‘prototypical’ plate is shown by averaging the row and column values for a large number of plates and replicates (Fig. 2; n = 24 plates). Since an underlying assumption of our screen is that the majority of the extracts would not have specific effects on the parameters we investigated and since the extracts are placed randomly on the plates, it is highly unlikely that the deviations seen in the outside rows and columns represent true hits.

A powerful method for mitigating row and column effects is known as the B score transformation. The median-based B score is analogous to the mean-based Z score: an adjusted numerator is divided by a measure of dispersion [44]. Being median based, the B score is less influenced by the effects of outliers (e.g. hits) and makes no assumptions about the distribution of the data [45]. An additional benefit of the B score is its ability to easily accommodate excluded data points, a common statistical issue. B score transformation successfully mitigated row and column effects found in the raw data (Figs. 2 and S4). Kruskal-Wallis tests of each example indicate a significant difference between the un-normalized medians of rows and columns (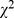 = 20.36, df = 7, *p* = 0.004; 3B: 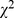 = 29.811, df = 11, *p* = 0.001; 3C: 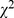 = 61.27, df = 7, *p*<0.001; 3D: 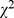 = 20.9, df = 11, *p* = 0.03). Application of the B score transformation resulted in a lack of significant differences found between the row and column medians (Kruskal-Wallis: 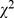 = 0.91, df = 7, *p* = 0.996; 4B: 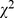 = 1.29, df = 11, *p* = 0.999; 4C: 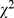 = 2.08, df = 7, *p* = 0.955; 4D: 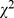 = 2.75, df = 11, *p* = 0.993). B score transformation of the data did not result in a symmetrical distribution of the data within a row or column but generally left the relative ranking of extracts intact (Fig. S5).

### Hit Selection

**Figure 3:**
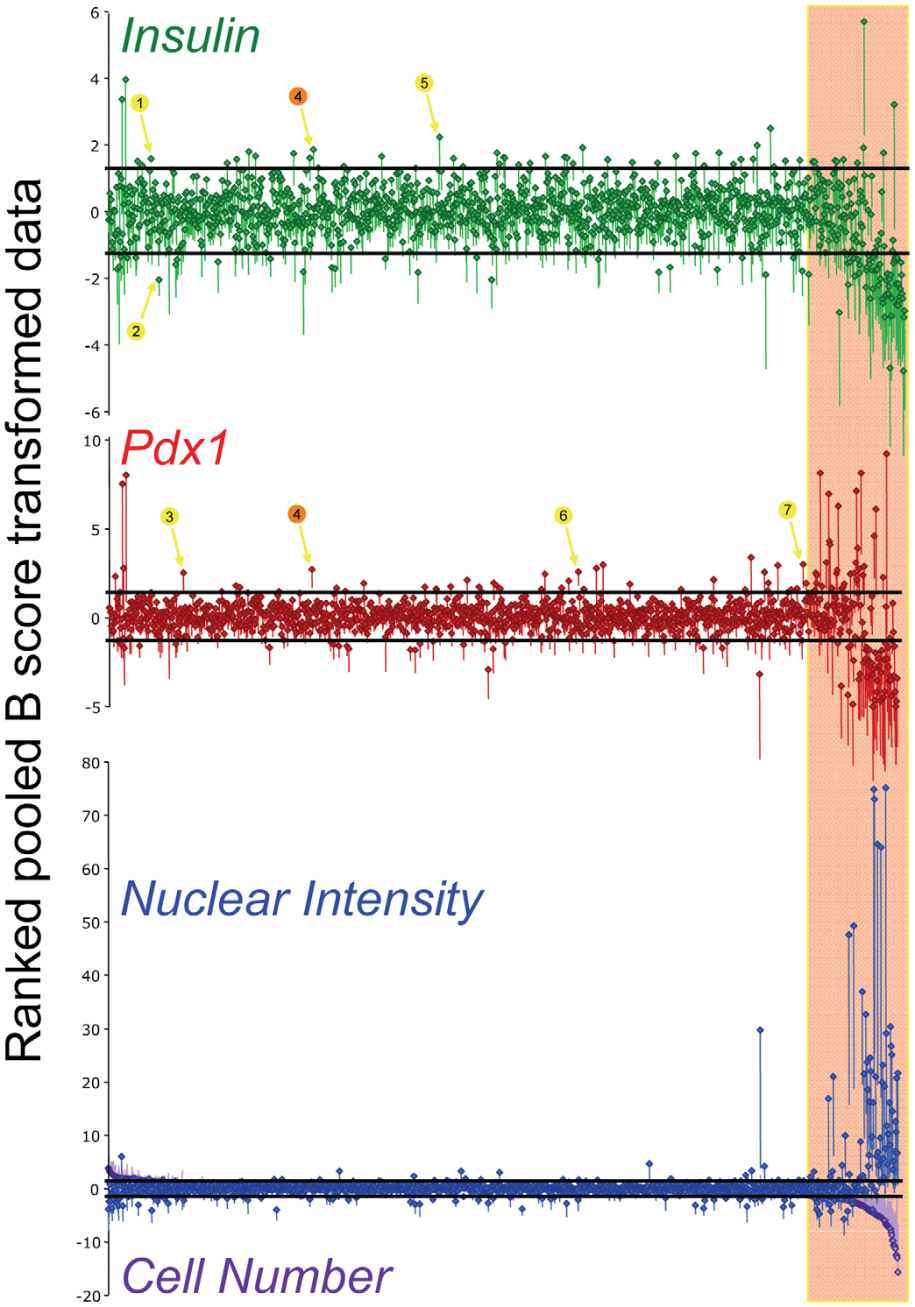
Results from multi-parameter high-content screening of marine invertebrate extract library. B-score normalized values are shown for *insulin* activity (green), *pdx1* activity (red), nuclear DNA staining intensity (blue), and cell number per well (purple). In each panel, the extracts are ranked according to their effect on cell count (bottom panel). Data are from five independent screens conducted on the MIN6 beta-cell line. Black lines around the center median indicate the median +/− 2*(Median Absolute Deviation; MAD), a measure of the spread of the data, analogous to the standard deviation. Hits (numbered circles and arrows) were defined as any extract whose SEM fell outside of the range of the median +/− 2*MAD. DMSO controls have been omitted from the data. Images with obvious artifacts (e.g. fibers) were omitted from data analysis.

We next sought to determine which extracts altered each specific cellular parameter. First, results were ranked and plotted by cell number (Fig. 3). This representation of the data clearly shows that approximately 15% of extracts at their initial doses were cytotoxic, that is yielding a low cell number and intense Hoechst fluorescence. Nuclear DNA condensation is a well-established hallmark of the apoptotic cell death pathway. After excluding these extracts, we calculated 2 median absolute deviations from the median B-score. Extracts that had a standard error of the mean that did not overlap with these lines were considered significant hits. Analysis of these hits in isolation shows that extracts that affect *insulin* gene expression typically also modulated the expression of *pdx1* promoter activity. We compared the results with the MIN6 cells (5 replicates) with a single screen using 697 of the same extracts performed on human islet cells (Fig. S6). A handful of other pilot experiments with human islets were conducted, but issues of low beta-cell purity and insufficient lentiviral infection rates meant that most of the replicates were un-interpretable. In the one pilot study in which we obtained a relatively high percentage of labeled beta-cells, *Insulin* and *pdx1* promoter activity were weakly correlated, with R^2^ values of 0.027 and 0.010, respectively. A stronger degree of correlation was observed between compounds that affected MIN6 cell death and human islet cell nuclear intensity and cell number, with R^2^ values of 0.067 and 0.077, respectively (Fig. S6). Together, these data demonstrate the feasibility of multi-parameter high-content screening for regulators of beta-cell fate and function. Further work will be required to increase the lentiviral infection rate and beta-cell purity before this high-content screening platform can be routinely applied to human islet cultures.

### Hit Validation and Compound Purification

Out of 1319 extracts, we narrowed our interest to 7 (Table 1). We focused our attention on hits that affect the promoter activity of *insulin* (GFP, Channel 2) and *pdx1* (RFP, Channel 3), while ignoring for the present time significant changes in nuclear dye intensity (Hoechst, Channel 1). An extract derived from a sponge from Papua New Guinea (hit #4 in Fig. 3) caused significant enhancement of both *insulin* and *pdx1* promoter activity. We also found that a sponge extract from Indonesia (Menado) (hit #1) and a sponge extract from Papua New Guinea (hit #5) significantly increased *insulin* promoter activity. A sponge extract from Papua New Guinea (hit #2) significantly reduced *insulin* promoter activity. Extracts from a sea cucumber echinoderm from Indonesia (hit #6), a sea cucumber echinoderm from Pohnpei (hit #3), and a sponge from Indonesia (hit #7) increased *pdx1* promoter activity.

**Table 1:** B scores in all four parameters for each significant extract.

Hit #	Order	Details	Insulin	SEM	Pdx	SEM	Hoechst	SEM	Cell #	SEM
1	71	sponge from Indonesia (Menado)	1.56*	0.06	1.38	0.39	0.94	0.46	1.20	0.78
2	84	sponge from Papua New Guinea **#**	−2.06*	0.48	−0.77	0.68	−1.86	1.22	1.13	1.04
3	125	sea cucumber echinoderm from Pohnpei **#**	0.12	0.19	2.50*	1.06	0.90	0.36	0.93	0.52
4	340	sponge from Papua New Guinea	1.84*	0.50	2.69*	1.05	1.13	0.64	0.41	0.53
5	549	sponge from Papua New Guinea	2.21	1.11	1.45	1.59	0.44	0.56	0.12	0.32
6	786	sea cucumber echinoderm from Indonesia	1.90	0.77	2.55*	0.77	1.55	0.58	−0.19	0.99
7	1162	sponge from Indonesia	0.31	1.17	2.96*	1.33	1.32	0.78	−1.22	1.12

It is critical to put the magnitude of these changes in insulin promoter activity into perspective. For example, we have recently conducted a low-throughput screen of 17 physiologically relevant growth factors and found changes in MIN6 cell and primary cell insulin gene expression and insulin promoter activity of 10–20% [26]. Since it is widely appreciated that insulin mRNA is more abundant in beta-cells that almost any other gene, multi-fold increases of total, stable insulin mRNA in adult, healthy beta-cells would not generally be expected. Screens designed to identify factors that can increase insulin expression should therefore be adequately powered and possess multiple biological replicates. To further add context to our screening data, we treated glucose-responsive MIN6 cells with either 5 mM glucose or 10 mM glucose (Fig. S7A), a modest step in the physiological range of a beta-cell. Indeed, we confirmed that our MIN6 cell cultures are responsive to a step to 10 mM glucose using Fura-2 calcium imaging (Fig. S7B). Thus, the magnitude of changes in insulin promoter activity seen in our hits and the increases in insulin gene expression observed upon follow-up (below) should be considered robust and physiologically significant.

**Figure 4:**
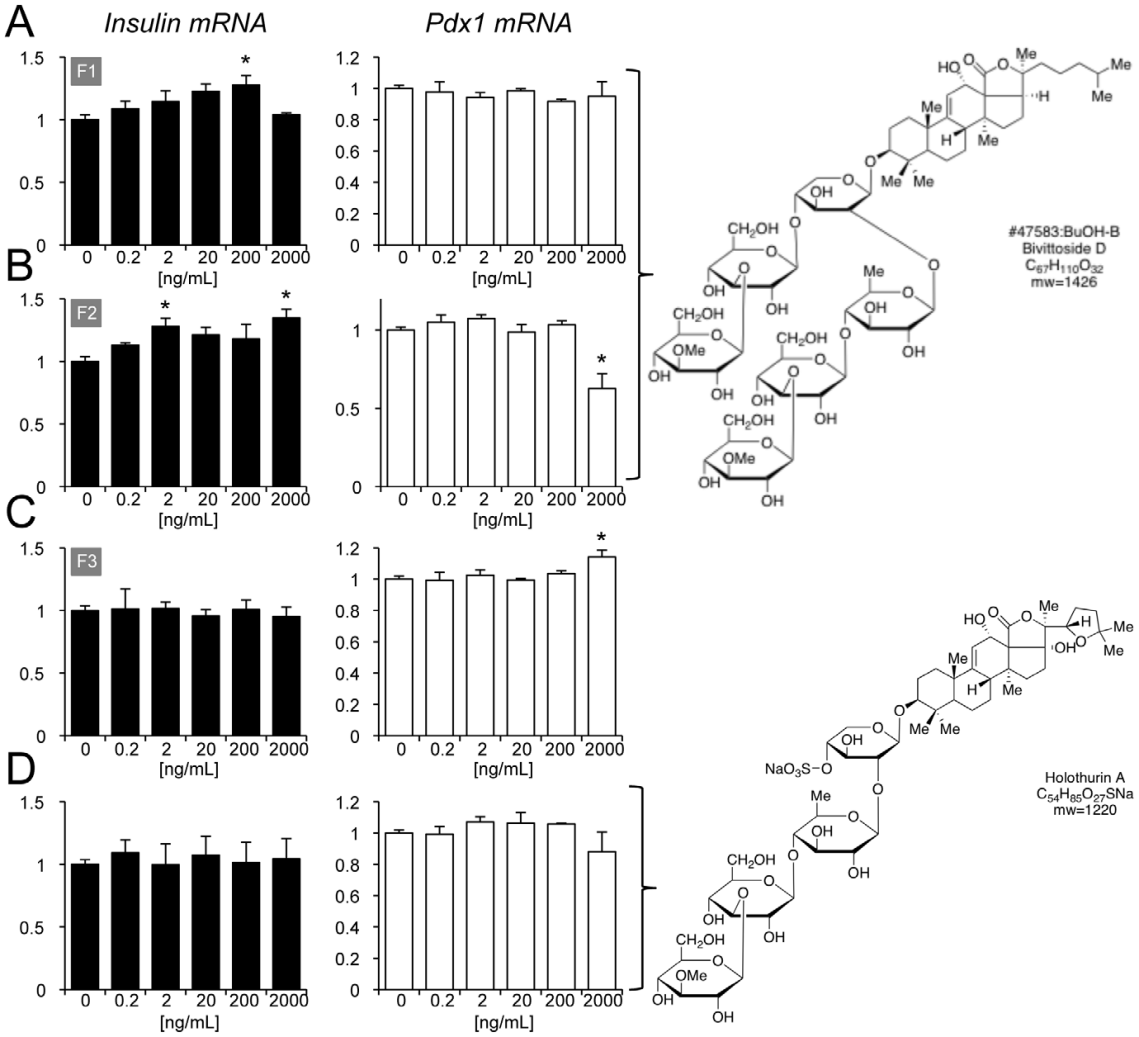
Identification of Bivittoside D as a positive regulator of insulin gene expression. (**A–C**) MIN6 cells were treated for 18 hours with multiple doses (0.2 to 2000 ng/mL) of the three purified fractions (F1, F2, F3) from the sponge extract identified as hit #3. (**D**) Cells were also treated with Holothurin A, which is found in similar sponges. RNA was isolated and quantitative qRT-PCR was conducted for Ins1 and Pdx1. Results were normalized to housekeeping genes (cyclophilin and beta-actin). n = 3. * P<0.05 compared to DMSO treated controls.

Secondary validation is required for any screening endeavor. First, we repeated the image-based, multi-parameter screen with the 7 hits described above, each at 3–5 doses (data not shown). However, promoter activity can theoretically be uncoupled from mRNA levels for a specific gene. Thus, we turned to real-time PCR to study the effects of specific compounds on mRNA levels of the *insulin* and *pdx1* genes. For example, hit #3 increased total insulin mRNA levels to an impressive degree, given the fact that insulin message is one of the most abundant transcripts in MIN6 cells (Fig. S8). We followed hits based on availability of the extracts and began with those found to have the least amount of cellular toxicity in the primary screen (i.e. lower Hoechst B scores in Table 1). Hit #3, sea cucumber echinoderm from Pohnpei, was further fractionated into pure compounds that were tested for their effects on insulin and Pdx1 mRNA (Fig. 4A–C). Fractions A and B, comprising Bivittoside D, showed an increase in insulin mRNA using TaqMan real-time PCR at doses as low as 2 ng/ml. The effects of Bivittoside D were quite specific because the related chemical holothurin A did not increase insulin gene expression (Fig. 4D). The fractions from extract #3 did not have consistent effects on chronic insulin secretion, which is an index for cell viability, except at the highest dose (Fig. S9).

It is also of considerable interest to identify factors that interfere with insulin gene expression because these drugs can then be used to identify the underlying mechanisms of gene expression in the beta-cell. Therefore, we also examined hit #2, from a sponge found in Papua New Guinea, which was identified as a negative regulator of insulin gene expression in the original screen. We observed strong inhibitory effects of fractions from this extract on insulin and Pdx1 promoter activity and beta-cell survival (Fig. S10). This was accompanied by a strong dose-dependent inhibition of chronic insulin secretion from treated MIN6 cell cultures (Fig. S11).

## Discussion

In this report, we present a novel imaging-based screening platform designed to identify compounds capable of altering the differentiation state, survival and/or proliferation of pancreatic beta-cells. To the best of our knowledge, this is the first implementation of multi-parameter, high-content, high-throughput screening in living pancreatic beta-cells. We also describe data analysis and statistical approaches that eliminate spurious results arising from row and column biases that are systematic in multi-well live cell analysis. We present results from multiple replicate screens that highlight several extracts capable of significantly altering *insulin* and *pdx1* promoter activity in beta-cells. We identified the active insulin gene expression promoting component, Bivittoside D, from one of these extracts.

The key elements in successful high-throughput screening programs are the judicious choice of validated molecular targets and access to the greatest number and structural diversity of compounds. Roughly four decades of investigations into natural products derived from marine invertebrates have shown that they are an exceptionally rich source of novel chemotypes that frequently exhibit potent biological activities. Marine sponge extracts also capture some of the chemical diversity of the associated microorganisms. Sponges are a rich source of terpenoids, peptides, alkaloids, polyketides, glycolipids, steroids, and many compounds with mixed biogenetic origins. Notably, this library of compounds has already produced several drug candidates for cancer therapy [46], [47], [48], [49]. In the present study, the use of multiple parameters was extremely valuable, as it allowed us to eliminate many hits from compounds that were also cytotoxic and would therefore be unsuitable leads for improving beta-cell function. The resulting seven lead extracts showed no effect on cell number or nuclear staining intensity in the primary screen. In the future, it might also be useful to investigate other methods of hit selection, including those that simultaneously weigh effects using multiple parameters.

From the selected seven extracts detected in the primary screen, we followed up on two extracts that had a positive and negative effect on insulin expression. From our secondary screens and follow-up studies, Bivittoside D was identified as a positive regulator of insulin gene expression. Bivittoside D is a lanostane triterpenoid with six monosaccharide units. Bivittoside D collected from fish has been shown to have spermicidal properties at high doses [50], however our fractions containing Bivittoside D had no cytotoxic effects at much lower doses and increased insulin expression at doses as low as 2 ng/ml. These differing effects demonstrate the breadth of biological activities available for discovery by screening of extracts from marine animals.

High content screening generates a more comprehensive cellular phenotype and therefore a more effective way to determine hits. However, these large datasets make data analysis challenging thus we opted to collapse thousands of single cell measurements into a ‘per well’ measurement. While any summarization results in the loss of information, the ‘per well’ measurement includes cell morphology information that is unavailable to traditional high-throughput screening, and the image data can be later mined for further insights. Examining the median intensity value minimizes the impact of any outlying cell intensities, while the 99^th^ quantile allows us to examine the extreme values, which are relevant in a mixed population of (mostly non-responding) cells.

Plate-based assays have inherent screening challenges such as systematic row and column errors. We have developed methods for systematic cell identification and summarization of relatively homogeneous cultures (MIN6 cells) and heterogeneous cultures (human pancreatic endocrine tissue), as well as statistical measures for mitigating plate-based artifacts on the effects of natural extracts on cell proliferation, apoptosis and expression of *insulin* and *pdx1* in these cells. In our studies, the significant row and column effects were alleviated by B score normalization. One shortcoming of the B score is its inability to account for synergistic row and column effects due to the additive nature of the median polish inherent in its calculation, which can lead to both type I and type II error. This is minimized experimentally by repeated measurements and secondary analysis. Further analysis of extracts of interest will focus on single cell analysis, to ensure that the observed effects are cell-autonomous.

In summary, we have conducted an automated imaging screen of 1319 extracts isolated from marine invertebrates collected from around the world using human islet cells and MIN6 cells. We have optimized parameters for both screening and image analysis. Hopefully, the identification of other novel natural products that significantly modulate *insulin* and/or *pdx1* promoter activity will point to new methods of controlling beta-cell function and fate and eventually to new diabetes therapeutics based on the lead structures of these natural products.

## Supporting Information

Methods S1 Supplemental Methods.
(0.09 MB DOC)

Figure S1 High-throughput, high-content experimental design. Shown are the timelines for both MIN6 cell experiments and experiments performed on dispersed human islets cells.
(0.05 MB PDF)

Figure S2 Object identification and segmentation. Example images of Hoechst-treated human pancreatic tissue (A, B, C) and MIN6 cells (D, E, F) acquired by the Cellomics ArrayScan VI. Raw images (A, D) were subjected to the Cellomics Target Activation BioApplication's nuclear identification (B, E) and cytoplasmic masking (C, F). Blue indicates objects that are selected given user-specified criteria (see results), orange indicates objects that do not meet selection criteria and green indicates the area of the cytoplasmic mask. (G–I) Comparison of non-optimal and optimal segmentation using Cellomics Target Activation.
(1.34 MB PDF)

Figure S3 Example row and column biases in 96-well format high-throughput data. Examples of row (A, B) and column (C, D) effects in Hoechst expression of human pancreatic tissue (A, C) and in cell number in MIN6 (B, D) data of individual plates. Row and column effects are generally more pronounced in the human data. Lower expression levels in the perimeter wells is were typical (A, B, D), although occasionally expression levels are were reduced in non-perimeter rows or columns (C, column 8).
(0.08 MB PDF)

Figure S4 B score transformation corrects row and column biases in individual plates. Row (A, B) and column (C, D) effects in Hoechst expression of human pancreatic tissue (A, C) and in cell number in MIN6 (B, D) data after B score transformation. These data correspond to the data in Figure S3.
(0.08 MB PDF)

Figure S5 B score transformed data compared with raw data. Correlations between median values per well (X-axis) and B-score transformed values per well (Y-axis) for each extract for each of the 4 parameters analyzed.
(0.14 MB PDF)

Figure S6 Correlation of MIN6 data with a single multi-parameter high content screen on human islet cell cultures. B scores are shown for a subset of the data illustrated in Figure 3 and a corresponding data set compiled from human islet cells exposed to the same extracts. Correlations are shown.
(0.23 MB PDF)

Figure S7 Effect of glucose on insulin promoter activity. (A) Control experiments with 5 mM glucose and 10 mM glucose demonstrating the magnitude of effects on insulin promoter activity under physiological conditions. Asterisks denote significant difference from DMSO control (n = 3). (B) Representative traces of Fura-2 loaded MIN6 cells exposed to 10 mM glucose from a basal glucose of 3 mM. Cells are subsequently shown to respond to direct depolarization with 30 mM KCl.
(0.10 MB PDF)

Figure S8 Real-time PCR analysis of Insulin and Pdx1 mRNA in MIN6 cells treated with Hit #3 crude extract. Effects of butanol extract and crude extract of hit #3, a sea cucumber echinoderm from Pohnpei (Original sample number# 47583 in the library). Asterisks denote significant difference from DMSO control (n = 4 for all experiments).
(0.12 MB PDF)

Figure S9 Effect of purified fractions from hit #3 and Holothurin A on insulin secretion in MIN6 cells. MIN6 cells were treated for 18 hours in DMEM 22.5 mM glucose containing media supplemented with 10% FBS and 0.2 to 2000 ng/mL purified sponge library extract #47583:BuOH-A (A), #47583:BuOH-B (B), #47583:BuOH-C (C), or Holothurin A (D). Media were collected and insulin levels were assayed with rat insulin RIA kit. n = 3, mean + SEM. * P<0.05 compared to DMSO treated.
(0.05 MB PDF)

Figure S10 Effects of purified fractions from hit #2 on Insulin and Pdx1 promoter activity in MIN6 cells. Insulin promoter activity (green), Pdx1 promoter activity (red), and total cell number (purple) was analyzed for 5 doses of crude, butanol, aqueous extracts of hit #2, as well as four ethanol purified fractions from this extract (A–D).
(0.14 MB PDF)

Figure S11 Effect of crude extract of hit # 2 on insulin secretion from MIN6 cells. MIN6 cells were treated for 24 hours in serum free media supplemented with 1∶1, 1∶10, 1∶100, 1∶1000 dilutions of the hit #2 sponge extract (original library number #03-436). Media was collected and insulin levels were assayed with rat insulin RIA kit. n = 5, mean ± SEM.
(0.07 MB PDF)
