# Supplementary material for: A Multi-Parameter, High-Content, High-Throughput Screening Platform to Identify Natural Compounds that Modulate Insulin and Pdx1 Expression
Source: PLoS One. 2010 Sep 23;5(9):e12958. doi: 10.1371/journal.pone.0012958 (PMC2944895; doi:10.1371/journal.pone.0012958)
Supplement: Methods S1 — Supplemental Methods. (0.09 MB DOC) [file pone.0012958.s001.doc]

**SUPPLEMENTAL METHODS**

*Lentiviral vectors*

A third generation feline immunodeficiency virus (FIV) vector based on pTiger was kindly provided by Dr. Garry Nolan (Stanford, CA) and modified to contain a 410 base pair rat *Insulin I* (*Ins1*) promoter-EGFP cassette and a 4530 base pair mouse *Pdx1* promoter-monomeric red fluorescent protein (mRFP) cassette as described in detail elsewhere (1). Detailed cloning information is available elsewhere (1). Virus production was performed as described (1, 2). Viral titers were consistently 1-4x10^6^ TU/ml. Single reporter vectors and *CMV* containing vectors were created as controls (1). Functional expression of each vector has been tested in HepG2, Panc-1, HEK293, MIN6 and INS-1 cell lines, as well as human and mouse islets (1). Infection efficiency, based on the percentage of cells infected with a CMV-mRFP virus, was 45 ± 4 % (average of 5 test plates).

*Human islet isolation and culture*

Human pancreata were isolated by the Ike Barber Clinical Islet Transplantion Laboratory at the University of British Columbia as described (3). Typically, islet preparations were 60-80% pure immediately after isolation, as assessed by dithizone staining. Human pancreatic islets were dispersed into single cells as described previously (4, 5), but with some modifications for the larger scale. For each donor pancreas, 0.5 mL packed cell volume (PCV) measured using PCV-tubes (Techno Plastic Product AG, Trasadingen, Switzerland) islet-rich human pancreatic tissue was centrifuged to create a dense pellet, then washed once in CMRL-FBS culture medium (CMRL, 10% FBS, 100 Units/mL Penicillin, 100 µg/mL Streptomycin, all from Invitrogen) and once in dispersion medium (Ca^2+^ and Mg^2+^ free HBSS, 1 mM EDTA, 10 mM HEPES, 0.5% BSA from Sigma, St. Louis, USA). The cell aggregates were re-suspended in a total of 10 mL dispersion medium and transferred to a 25 cm^2^ suspension T-flask. The cell aggregates were then incubated 7 minutes on a rotary shaker at 75 rpm at 37°C. Trypsin (Roche 109819) and DNase I (Sigma D5025) were added respectively to concentrations of 25 µg/mL and 4 µg/mL (8 Kunitz units/mL). After 10 minutes of digestion, mostly single cells and some clusters of cells were obtained and 20 mL CMRL-FBS were then added. The dispersed cell mixture was triturated 10-20 times and then passed through a 40 µm nylon mesh to remove remaining aggregates. Large aggregates of DNA and cell debris were discarded prior to sieving. The cell suspension (~120 million live cells, 70-80% viability) was centrifuged at 250 g for 7 minutes and seeded in CMRL-FBS at 40 000 cells/well in 96-well Viewplates (PerkinElmer) using a multichannel pipette. After 24 hours (day 1), all cells in sample wells for screening were infected with the dual reporter pTiger vector at an MOI of ~1.5. Some control wells pinned with DMSO were infected with the control CMV-eGFP or CMV-mRFP control pTiger vectors instead to assess infection efficiency. Non-adherent cells and the CMRL-FBS medium were carefully removed using a multichannel pipette. The concentrated virus stock was diluted in CMRL-SFM culture medium containing: phenol red-free CMRL (Cellgro), 0.2% bovine serum albumin (Stem Cell Technologies), 10 mM nicotinamide (Stem Cell Technologies), insulin (0.5 mg/L)/transferrin (0.5 mg/L)/selenium (5µg/L) (Sigma I1884, used at 1/10^th^ concentration), 100 Units/mL Penicillin and 100 µg/mL Streptomycin. An aliquot (100 μL) of this media was added to each well, followed by virus infection. On day 2, the virus-containing medium was replaced by 100 μL/well of fresh CMRL-SFM. Another medium exchange of 200 μL/well of fresh CMRL-SFM was performed on day 3 immediately prior to extract pinning.

*MIN6 cell culture*

MIN6 beta-cells stably expressing the lentiviral dual-reporter virus were cultured in DMEM media containing 25 mM glucose, phenol red, 10% fetal bovine serum, pencillin, and streptomycin (Invitrogen). Cells were plated at a density of ~6000-8000 cells per well in 96-well polystyrene plates (6)(ViewPlate, PerkinElmer). After a 24-hour incubation period, 100 μl DMEM was added to each well using a multi-well pipet to compensate for any media that had evaporated during the incubation period and reduce row and column effects prior to the addition of extracts. Cells were incubated at 37°C in CO_2_ controlled conditions unless otherwise noted.

*Extract library*

We screened stable dual reporter-expressing MIN6 cells and the human endocrine tissue fraction in the presence of a library of extracts derived from marine invertebrates, primarily sea sponges. Dried extracts were re-suspended in DMSO in 96-well plates and stored at -20°C. Details of this natural extract library have been published elsewhere (7). After a 24-hour incubation period, 100 μl DMEM was added to each well using a multi-well pipette to compensate for any media that had evaporated during the incubation period and reduce row and column effects. Immediately prior to pinning, extracts were thawed, covered in tin foil to minimize exposure to light and shaken for ~5 minutes at 550 rpm on an Orbital plate shaker. Extracts were transferred to plates by a pinning robot using 0.4 mm pins, transferring approximately 200 nl. After the addition of extracts, cells were incubated for 24 hours in the case of MIN6 cells or for 4 days (until day 7) in the case of human islets (Fig. S1).

*Image acquisition and object identification*

Prior to imaging, the high-glucose media were removed from cells and replaced with 100 μl per well 1x PBS and 2% FBS with 1:10000 Hoechst nuclear dye. Images were acquired by automated fluorescence microscopy using the Cellomics ArrayScan V^TI^ (Thermo Fisher). Images from each well were acquired in three separate fluorescent channels (for Hoechst, GFP and RFP), using a 10x objective. Object (cell) identification was performed using the Cellomics Target Activation algorithm, with background correction set to 10 pixels and with an isodata (peak-based) intensity threshold adjusted by -0.99 to refine object selection. Individual object resolution was aided by performing object segmentation based on indentations of objects. Human tissue samples have a greater tendency to aggregate when compared with MIN6 cells, so object segmentation was set to a greater value. Parameter values were optimized after testing a variety of combinations of values. Ultimately, the values selected minimized excluding objects with a trade-off in the inclusion of some cell aggregates for the exclusion of some single cells (Fig. S2). For each cell/object identified, average and total intensity and intensity variance measurements were taken.

*Image acquisition and object identification*

Images were acquired by automated fluorescence microscopy using the Cellomics ArrayScan V^TI^ (Thermo Fisher). Object (cell) identification was performed using the Cellomics Target Activation algorithm. For each cell/object identified, average and total intensity and intensity variance measurements were taken. Additional details are presented in the Supplement.

*B-Score Data Transformation*

Data were transformed to mitigate systematic row and column effects using the B-score method. It is calculated as follows:

$$Bscore=\frac{r_{ijp}}{1.4826*MAD_{p}}$$

The denominator term *MAD_p_* refers to the median absolute deviation of plate *p* and is calculated by:

where $r_{ijp}$ refers to the residual value of well (*i,j*) on plate *p.* Residuals are obtained by performing a two-way median polish on the data. This is an additive model that fits a common term per well and row and column effects plus any residual value (8). The median polish was calculated by finding and removing row and column medians in an alternating fashion until the sum of the absolute values of the residuals is less than a small value, here 10^-5^, or until a maximum number of iterations has occurred, here 200 (9). The residuals are expected to centre at zero, thus the magnitude of the median of the absolute values of the differences between each residual and the median residual value (*i.e.* *MAD_p_*) estimates the spread of the data. The residual is defined as follows:

where *y_ijp_* is the observed value in well (*i*, *j*) of plate *p*, and is the fitted value of that well, defined as the sum of the plate mean (), the row bias of row *i* () on plate *p* and the column bias of column *j* () on plate *p*. If data are normally distributed, *MAD_p_* can be made comparable to a standard deviation by multiplying by a scale factor, 1.4826 (10). Median and 99^th^ quantile values for each well were subjected to B score normalization. All B scores were calculated using the package *cellHTS* (9) for R or, subsequently, using code we have designed for R written by J. Hill.

*Compound Purification*

A specimen of the freeze-dried sea cucumber (161 g) collected in Pohnpei was extracted exhaustively with MeOH (3 x 100 mL) at RT. After evaporation the crude extract was partitioned between H_2_O (1x15 mL) and EtOAc (4x5 mL) followed by n-BuOH (4x5 mL). The combined dried active n-BuOH extract was then fractionated by Sephadex LH20 column chromatography to give an active fraction consisting of bivittoside D.

*Gene expression analysis*

MIN6 cells (passages 25-39) were maintained in high glucose DMEM (Invitrogen, Carlsbad, CA, USA) supplemented with 10 % FBS (Invitrogen, Carlsbad, CA, USA) and 1% penicillin/streptomycin (Invitrogen, CA). MIN6 cells were plated at 50-70% confluency in tissue culture treated 12 well plates, (NUNC, VWR, Mississauga, ON, Canada) and treated for 18-24 hours with 2 µl/ml of serially diluted (1x10^-1^, 1x10^-2^, 1x10^-3^) crude and butanol extracts in DMEM media. Total RNA was isolated by Trizol method (Invitrogen, Carlsbad, CA, USA) followed by RNA purification, using RNeasy kit, Qiagen (Mississauga, ON, Canada). DNase-treated total RNA (100ng) was converted to cDNA by qScript cDNA synthesis kit, (Quanta BioSciences, Inc., VWR, Mississauga, ON, Canada). Real-time PCR was performed on Applied Biosystems StepOnePlus™ platform. TaqMan custom design primers for Ins1 and Ins2 and cyclophilin N008907.1.pt were purchased from Integrated DNA Technologies (IDT, Coralville, IA). Sequences for primers and probes for Ins1 and Ins2 were provided by Dr. Jake Kushner (U Penn). Ins1 5’- GAA GTG GAG GAC CCA CAA GTG, probe /56-FAM/CCC GGG GCT TCC TCC CAG CT/3IABlk_FQ/, 3’ - ATC CAC AAT GCC ACG CTT CT, Ins2 5’ GAA GTG GAG GAC CCA CAA GTG probe /56-FAM/CCT GCT CCC GGG CCT CCA /3IABlk_FQ/, 3’ GAT CTA CAA TGC CAC GCT TCT G. Pdx-1 primers in SYBR green assay were: 5’ GCT CAC GCG TGG AAA GGC CAG T, 3’ AGC TGG CAG TGA TGT TGA ACT TGA CCG AGA G, β-actin primers 5’ TGC GTG ACA TCA AAG AGA AG, 3’ GAT GCC ACA GGA TTC CAT A. The relative changes in gene expression were analyzed by the 2^-∆∆^Ct method and statistics by one-way ANOVA (n=4-5, biological replicates).

*Insulin secretion*

MIN6 cells were treated with compounds, as indicated, for 18-24 hours in 25 mM glucose containing DMEM media supplemented with 10% FBS. Media were collected and centrifuged at 5000g for 5 minutes to remove any cellular debris and insulin levels were assayed with a rat insulin radioimmunoassay kit (Linco Research, St Charles, MO, USA).

*Statistics*

The R environment for statistical computing ([http://www.R-project.org](http://www.R-project.org/)) was used for data analysis during the screening process, unless otherwise indicated. In all cases, unless otherwise mentioned, five replicate experiments were performed and statistical tests were considered significant at an alpha level of 0.05. For follow-up studies, Graphpad Prism or Microsoft Excel were used to perform ANOVA or t-tests as appropriate. Results were considered significant if the *P* value was less than 0.05.

**REFERENCES**

1. **Szabat M, Luciani DS, Piret JM, Johnson JD** 2008 Maturation of Adult Beta-Cells Revealed Using a Pdx1/Insulin Dual Reporter Lentivirus. Endocrinology

2. **Curran MA, Nolan GP** 2002 Recombinant feline immunodeficiency virus vectors. Preparation and use. Methods Mol Med 69:335-350

3. **Warnock GL, Meloche RM, Thompson D, Shapiro RJ, Fung M, Ao Z, Ho S, He Z, Dai LJ, Young L, Blackburn L, Kozak S, Kim PT, Al-Adra D, Johnson JD, Liao YH, Elliott T, Verchere CB** 2005 Improved human pancreatic islet isolation for a prospective cohort study of islet transplantation vs best medical therapy in type 1 diabetes mellitus. Arch Surg 140:735-744

4. **Johnson JD, Misler S** 2002 Nicotinic acid-adenine dinucleotide phosphate-sensitive calcium stores initiate insulin signaling in human beta cells. Proc Natl Acad Sci U S A 99:14566-14571

5. **Luciani DS, Johnson JD** 2005 Acute effects of insulin on beta-cells from transplantable human islets. Mol Cell Endocrinol 241:88-98

6. **Luciani DS, Gwiazda K, Yang TL, Kalynyak TB, Bychkivska Y, Frey MH, Jeffrey KD, Sampaio AV, Underhill TM, Johnson JD** 2008 Roles of IP3R and RyR Ca2+ Channels in Endoplasmic Reticulum Stress and {beta}-Cell Death. Diabetes

7. **Tarling CA, Woods K, Zhang R, Brastianos HC, Brayer GD, Andersen RJ, Withers SG** 2008 The search for novel human pancreatic alpha-amylase inhibitors: high-throughput screening of terrestrial and marine natural product extracts. Chembiochem 9:433-438

8. **Tukey JW** 1977 Exploratory data analysis. Reading, Mass. ; Don Mills, Ont.: Addison-Wesley Pub. Co.

9. **Boutros M, Bras LP, Huber W** 2006 Analysis of cell-based RNAi screens. Genome Biol 7:R66

10. **Hoaglin DC, Mosteller F, Tukey JW** 1983 Understanding robust and exploratory data analysis. New York: Wiley
