# Supplementary figures and images for: A Multi-Parameter, High-Content, High-Throughput Screening Platform to Identify Natural Compounds that Modulate Insulin and Pdx1 Expression
Source: PLoS One. 2010 Sep 23;5(9):e12958. doi: 10.1371/journal.pone.0012958 (PMC2944895; doi:10.1371/journal.pone.0012958)

Figure S1

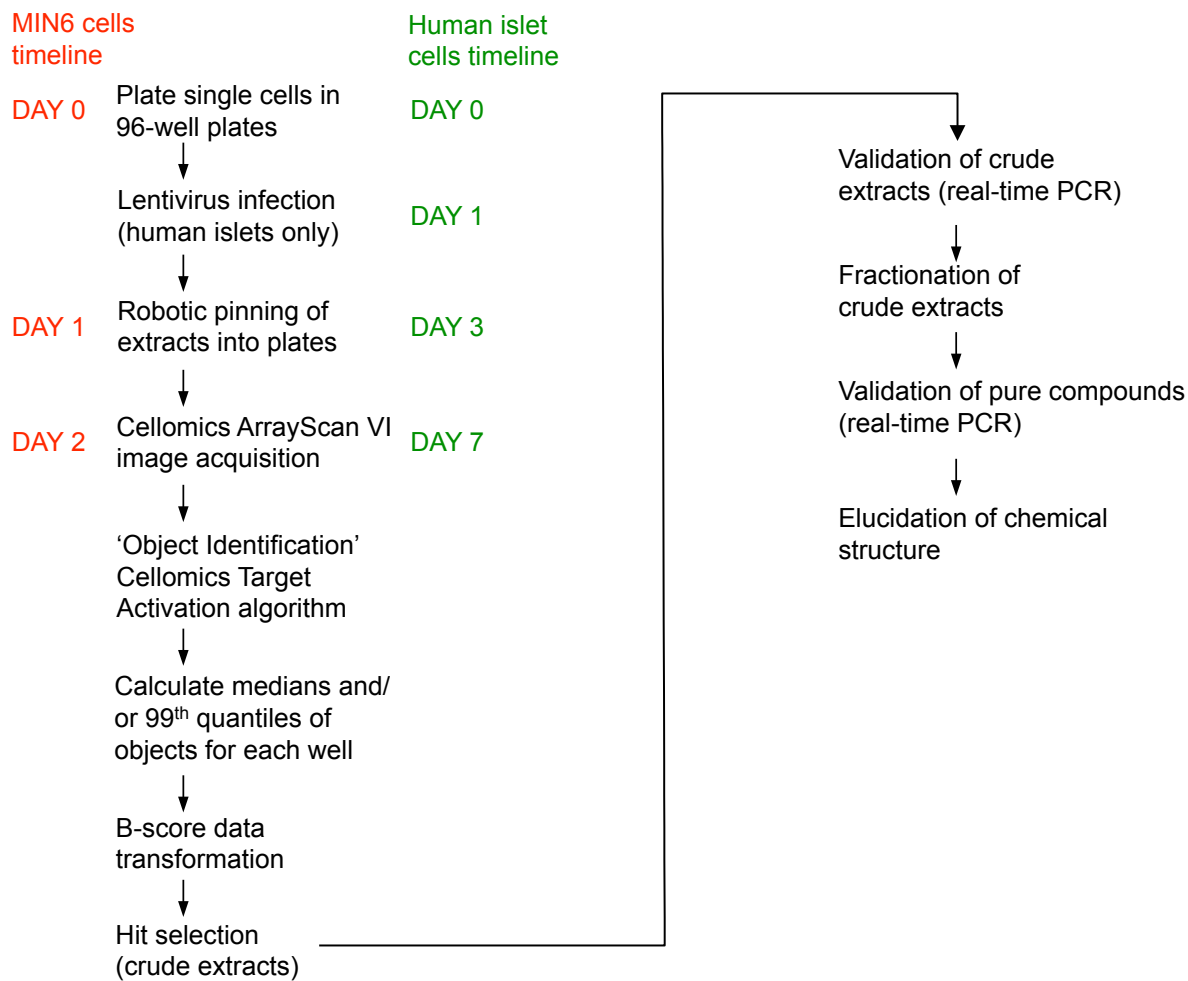

Supplement: Figure S1 — High-throughput, high-content experimental design. Shown are the timelines for both MIN6 cell experiments and experiments performed on dispersed human islets cells. (0.05 MB PDF) [file pone.0012958.s002.pdf]

Figure S2

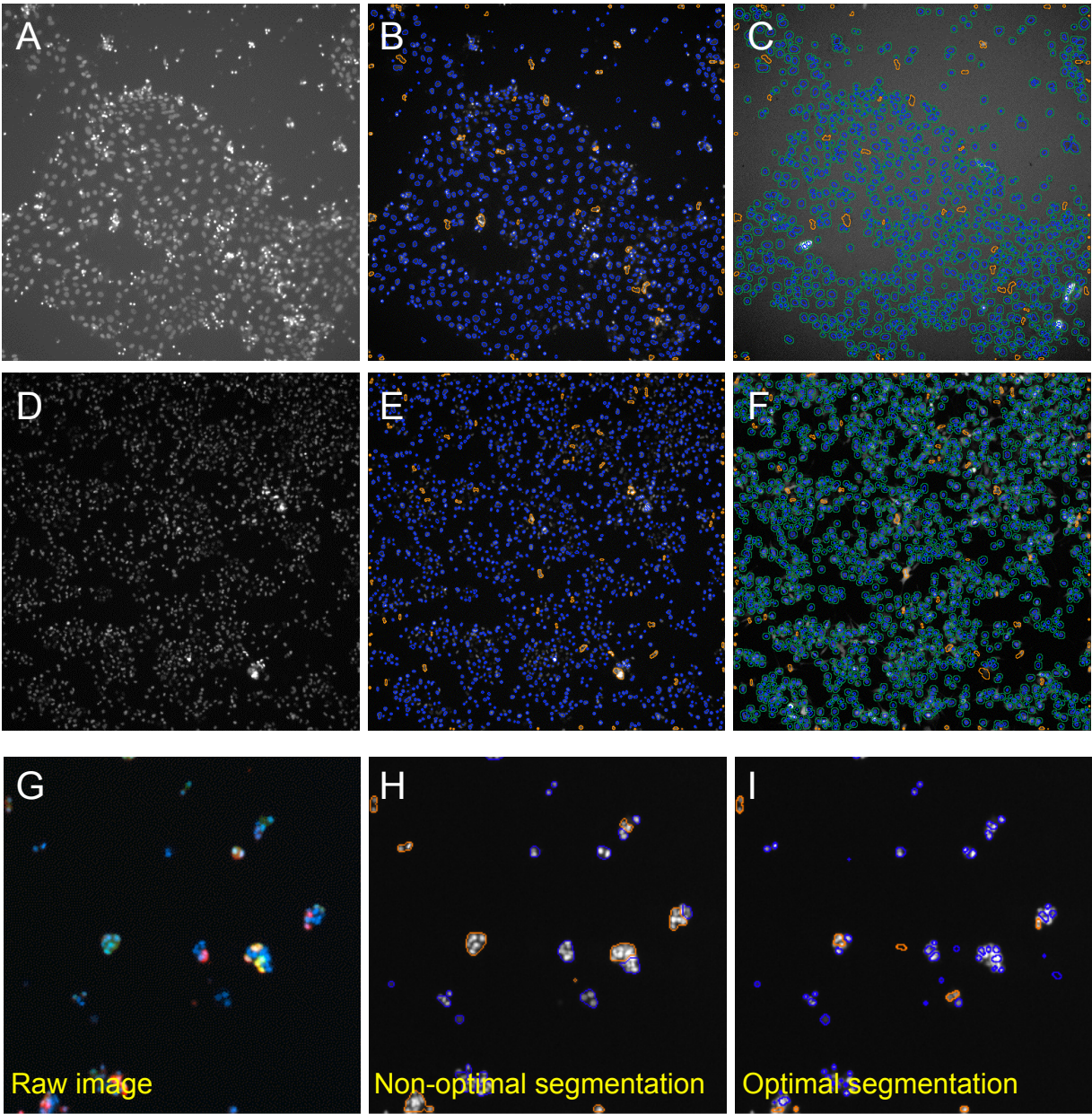

Supplement: Figure S2 — Object identification and segmentation. Example images of Hoechst-treated human pancreatic tissue (A, B, C) and MIN6 cells (D, E, F) acquired by the Cellomics ArrayScan VI. Raw images (A, D) were subjected to the Cellomics Target Activation BioApplication's nuclear identification (B, E) and cytoplasmic masking (C, F). Blue indicates objects that are selected given user-specified criteria (see results), orange indicates objects that do not meet selection criteria and green indicates the area of the cytoplasmic mask. (G–I) Comparison of non-optimal and optimal segmentation using Cellomics Target Activation. (1.34 MB PDF) [file pone.0012958.s003.pdf]

Figure S3

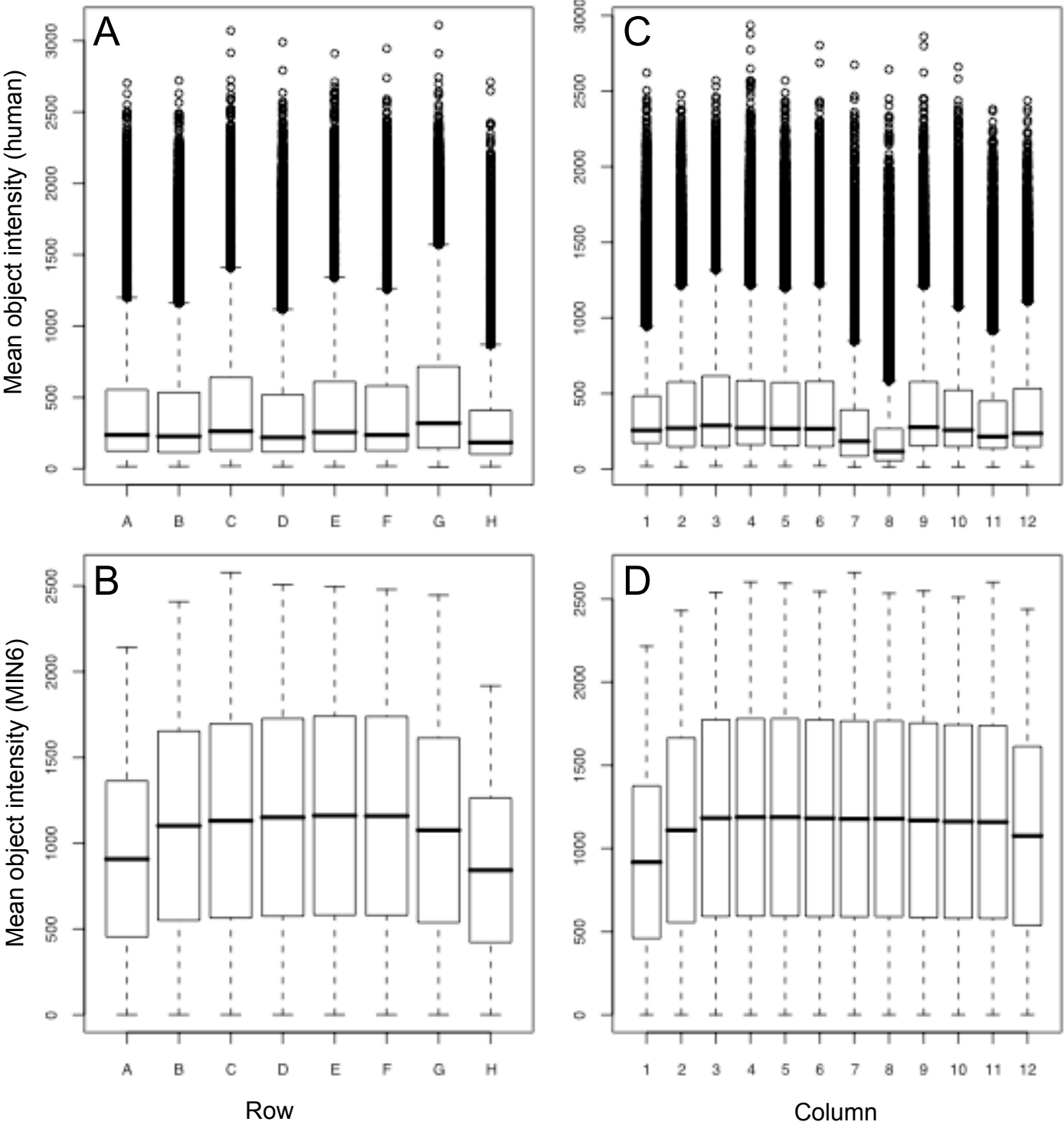

Supplement: Figure S3 — Example row and column biases in 96-well format high-throughput data. Examples of row (A, B) and column (C, D) effects in Hoechst expression of human pancreatic tissue (A, C) and in cell number in MIN6 (B, D) data of individual plates. Row and column effects are generally more pronounced in the human data. Lower expression levels in the perimeter wells is were typical (A, B, D), although occasionally expression levels are were reduced in non-perimeter rows or columns (C, column 8). (0.08 MB PDF) [file pone.0012958.s004.pdf]

Figure S4

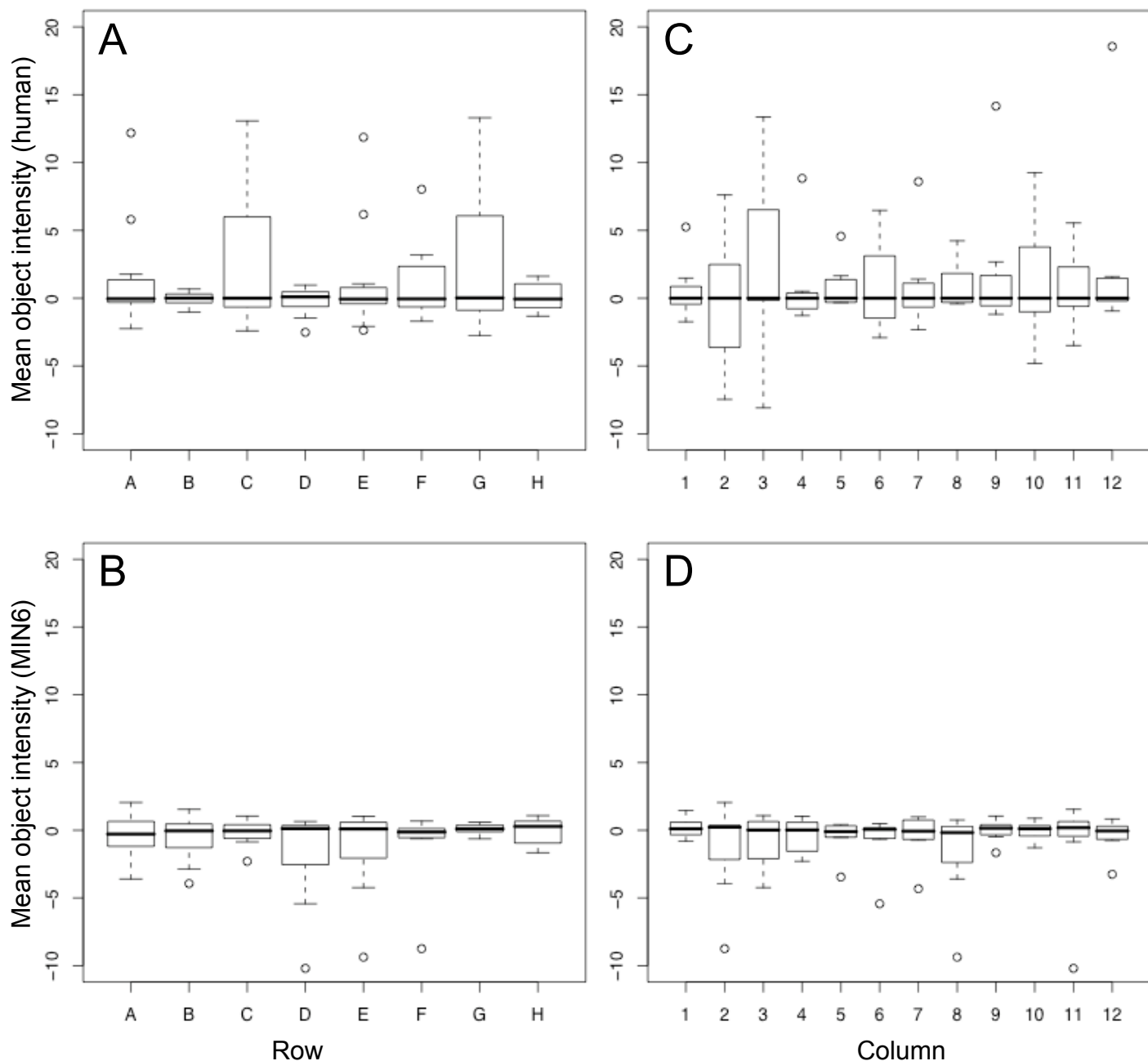

Supplement: Figure S4 — B score transformation corrects row and column biases in individual plates. Row (A, B) and column (C, D) effects in Hoechst expression of human pancreatic tissue (A, C) and in cell number in MIN6 (B, D) data after B score transformation. These data correspond to the data in Figure S3. (0.08 MB PDF) [file pone.0012958.s005.pdf]

Figure S5

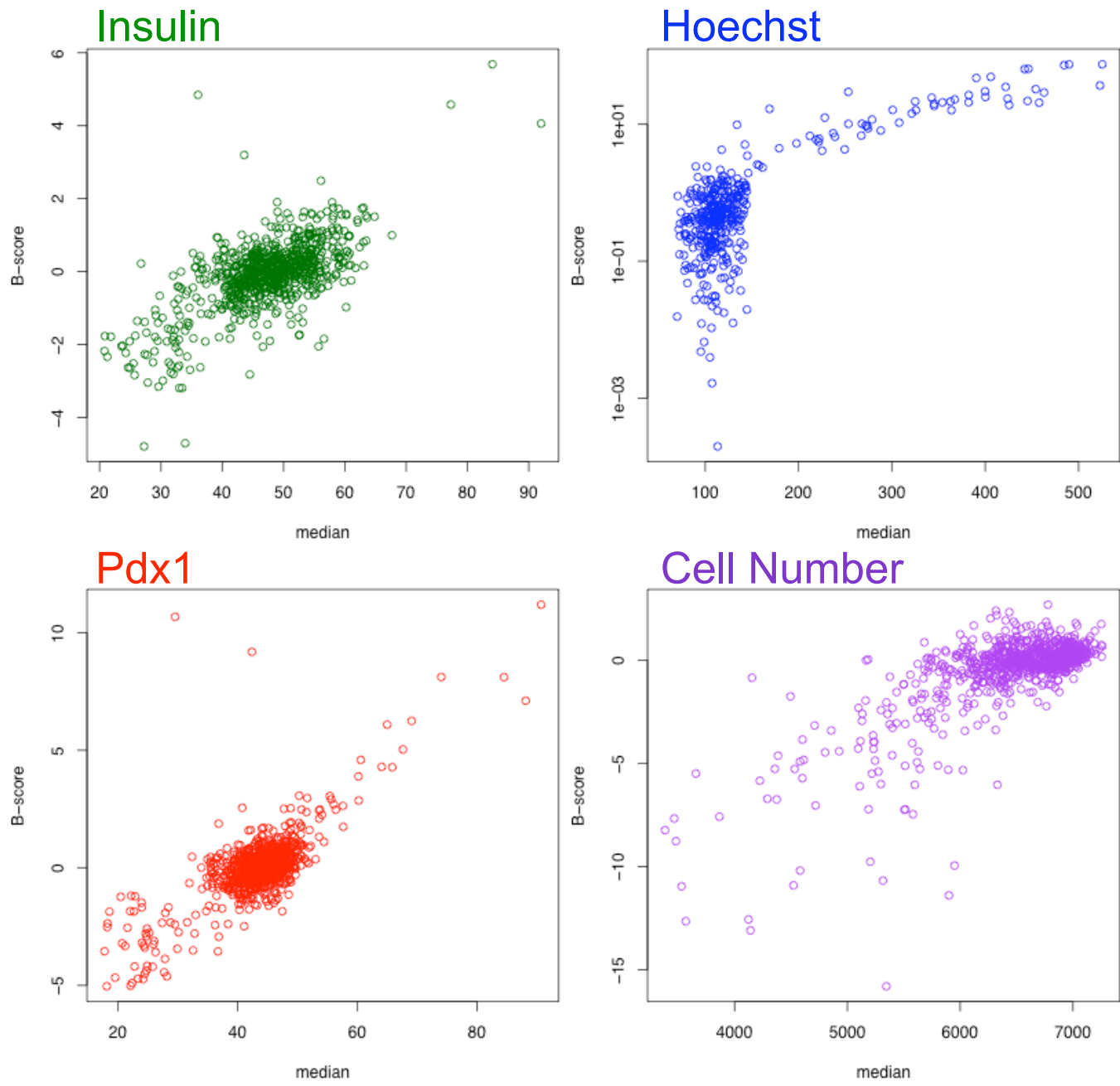

Supplement: Figure S5 — B score transformed data compared with raw data. Correlations between median values per well (X-axis) and B-score transformed values per well (Y-axis) for each extract for each of the 4 parameters analyzed. (0.14 MB PDF) [file pone.0012958.s006.pdf]

Figure S7

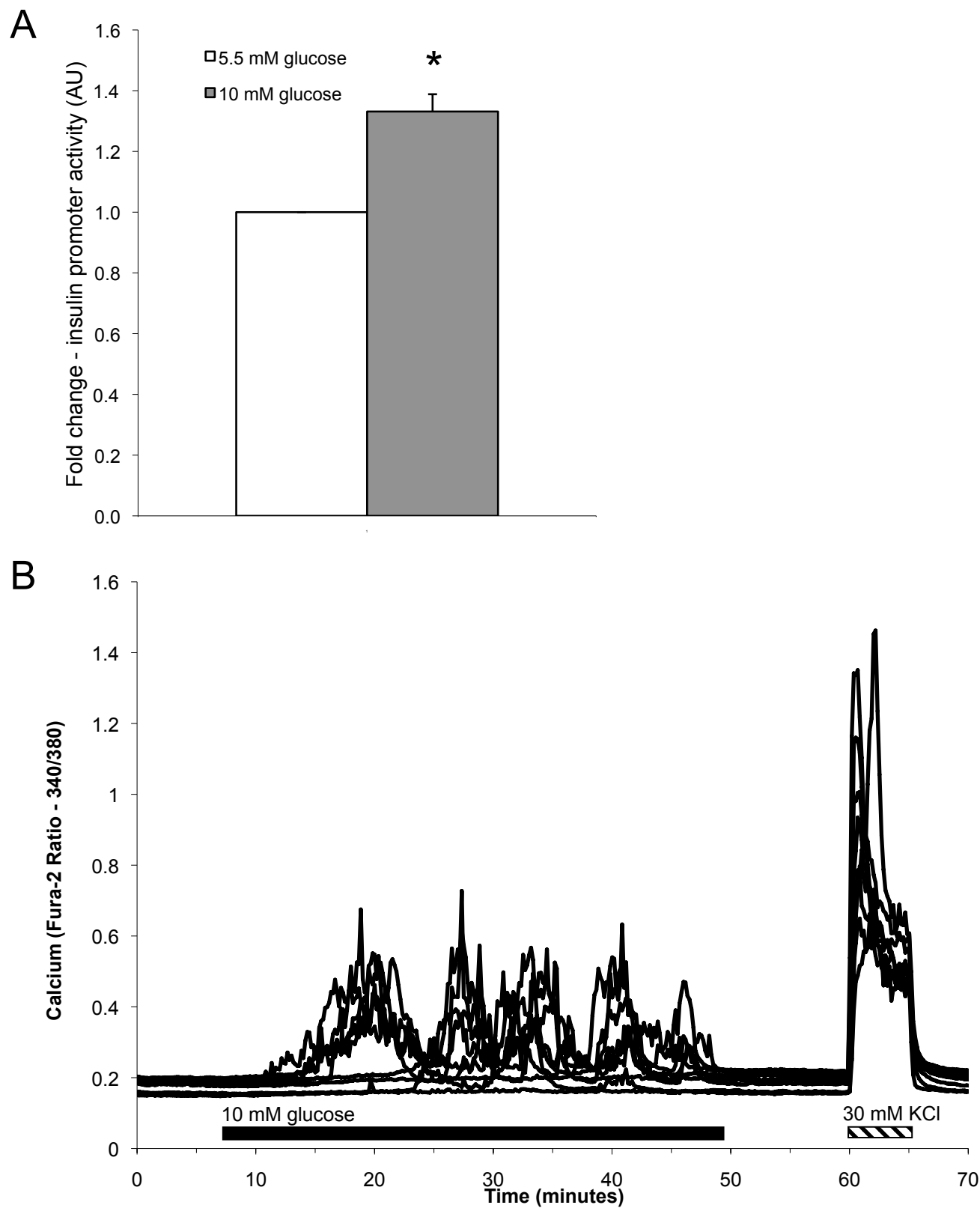

Supplement: Figure S7 — Effect of glucose on insulin promoter activity. (A) Control experiments with 5 mM glucose and 10 mM glucose demonstrating the magnitude of effects on insulin promoter activity under physiological conditions. Asterisks denote significant difference from DMSO control (n = 3). (B) Representative traces of Fura-2 loaded MIN6 cells exposed to 10 mM glucose from a basal glucose of 3 mM. Cells are subsequently shown to respond to direct depolarization with 30 mM KCl. (0.10 MB PDF) [file pone.0012958.s008.pdf]

Figure S8

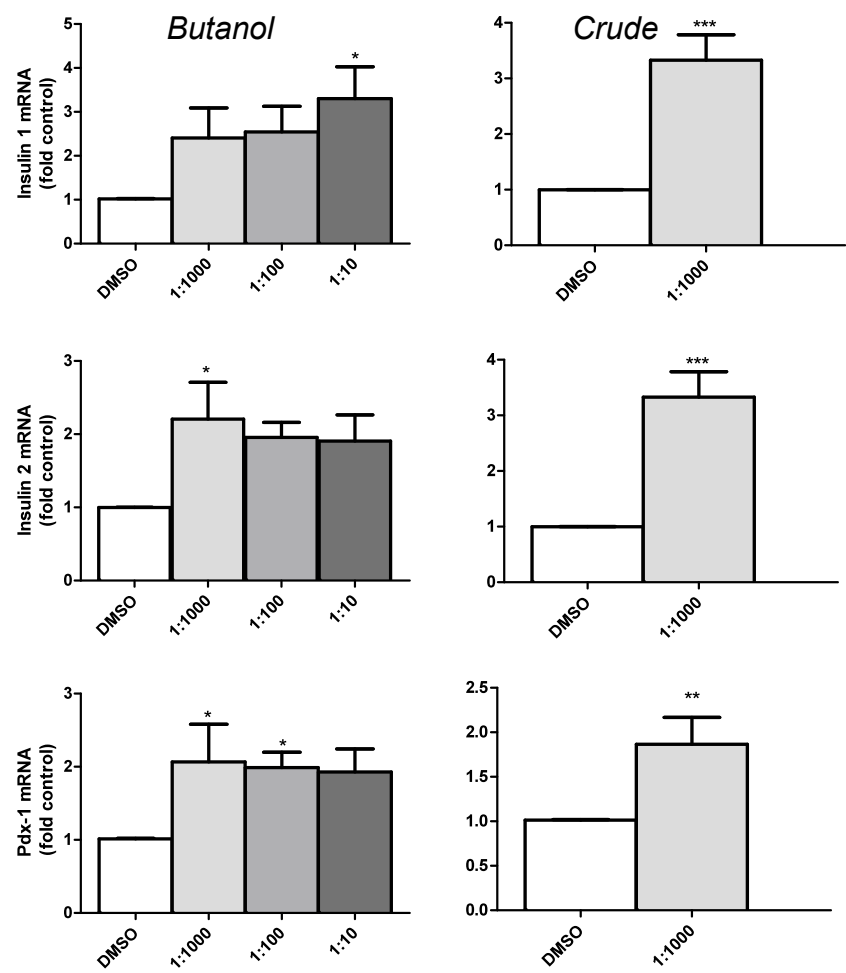

Supplement: Figure S8 — Real-time PCR analysis of Insulin and Pdx1 mRNA in MIN6 cells treated with Hit #3 crude extract. Effects of butanol extract and crude extract of hit #3, a sea cucumber echinoderm from Pohnpei (Original sample number# 47583 in the library). Asterisks denote significant difference from DMSO control (n = 4 for all experiments). (0.12 MB PDF) [file pone.0012958.s009.pdf]

Figure S9

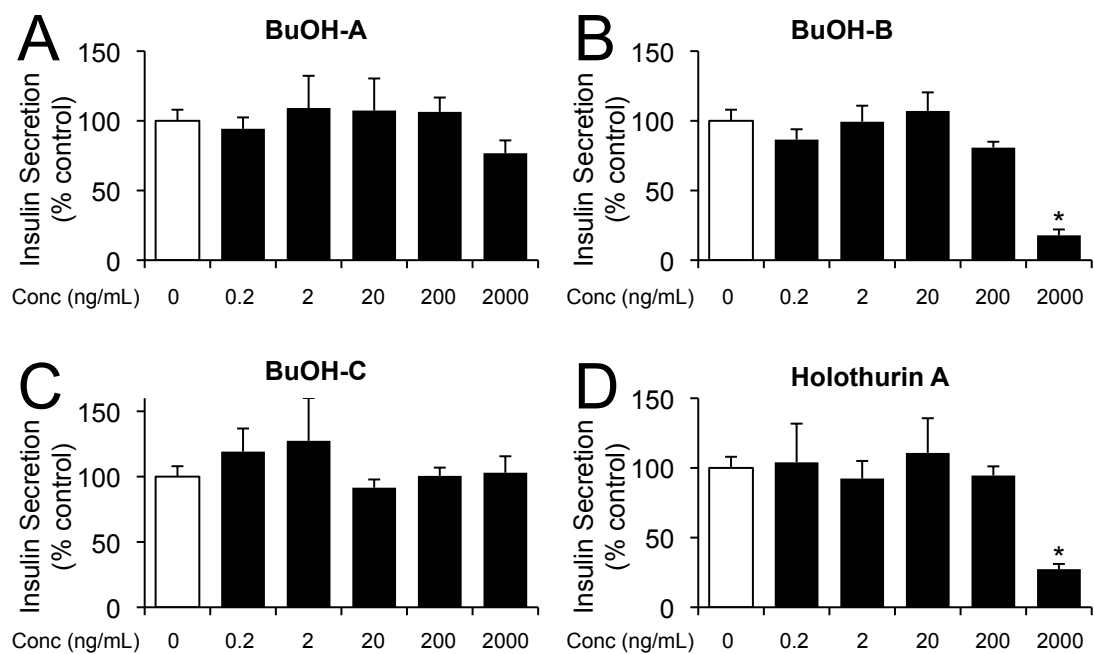

Supplement: Figure S9 — Effect of purified fractions from hit #3 and Holothurin A on insulin secretion in MIN6 cells. MIN6 cells were treated for 18 hours in DMEM 22.5 mM glucose containing media supplemented with 10% FBS and 0.2 to 2000 ng/mL purified sponge library extract #47583:BuOH-A (A), #47583:BuOH-B (B), #47583:BuOH-C (C), or Holothurin A (D). Media were collected and insulin levels were assayed with rat insulin RIA kit. n = 3, mean + SEM. * P<0.05 compared to DMSO treated. (0.05 MB PDF) [file pone.0012958.s010.pdf]

Figure S10

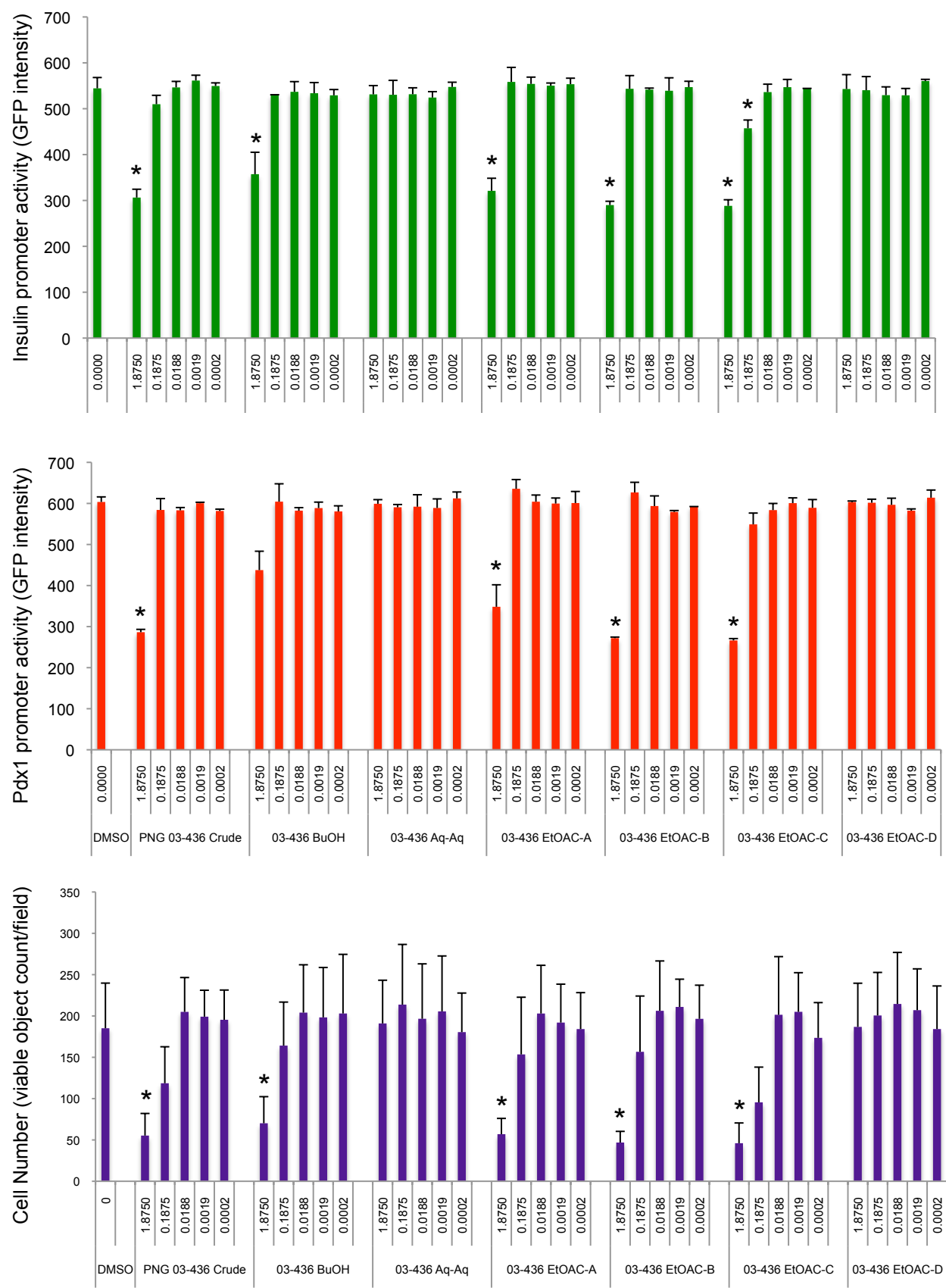

Supplement: Figure S10 — Effects of purified fractions from hit #2 on Insulin and Pdx1 promoter activity in MIN6 cells. Insulin promoter activity (green), Pdx1 promoter activity (red), and total cell number (purple) was analyzed for 5 doses of crude, butanol, aqueous extracts of hit #2, as well as four ethanol purified fractions from this extract (A–D). (0.14 MB PDF) [file pone.0012958.s011.pdf]

Figure S11

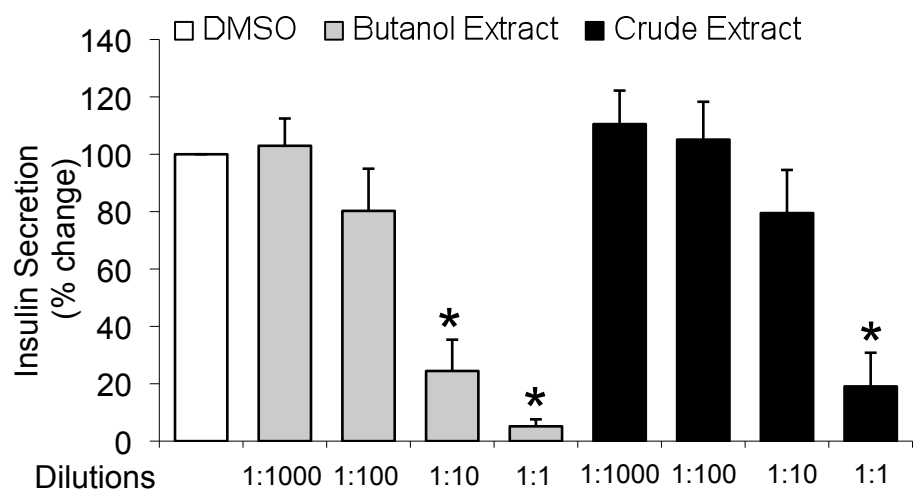

Supplement: Figure S11 — Effect of crude extract of hit # 2 on insulin secretion from MIN6 cells. MIN6 cells were treated for 24 hours in serum free media supplemented with 1∶1, 1∶10, 1∶100, 1∶1000 dilutions of the hit #2 sponge extract (original library number #03-436). Media was collected and insulin levels were assayed with rat insulin RIA kit. n = 5, mean ± SEM. (0.07 MB PDF) [file pone.0012958.s012.pdf]
