# Supplementary material for: A Multi-Parameter, High-Content, High-Throughput Screening Platform to Identify Natural Compounds that Modulate Insulin and Pdx1 Expression
Source: PLoS One. 2010 Sep 23;5(9):e12958. doi: 10.1371/journal.pone.0012958 (PMC2944895; doi:10.1371/journal.pone.0012958)
Supplement: Figure S6 — Correlation of MIN6 data with a single multi-parameter high content screen on human islet cell cultures. B scores are shown for a subset of the data illustrated in Figure 3 and a corresponding data set compiled from human islet cells exposed to the same extracts. Correlations are shown. (0.23 MB PDF) [file pone.0012958.s007.pdf]

Figure S6

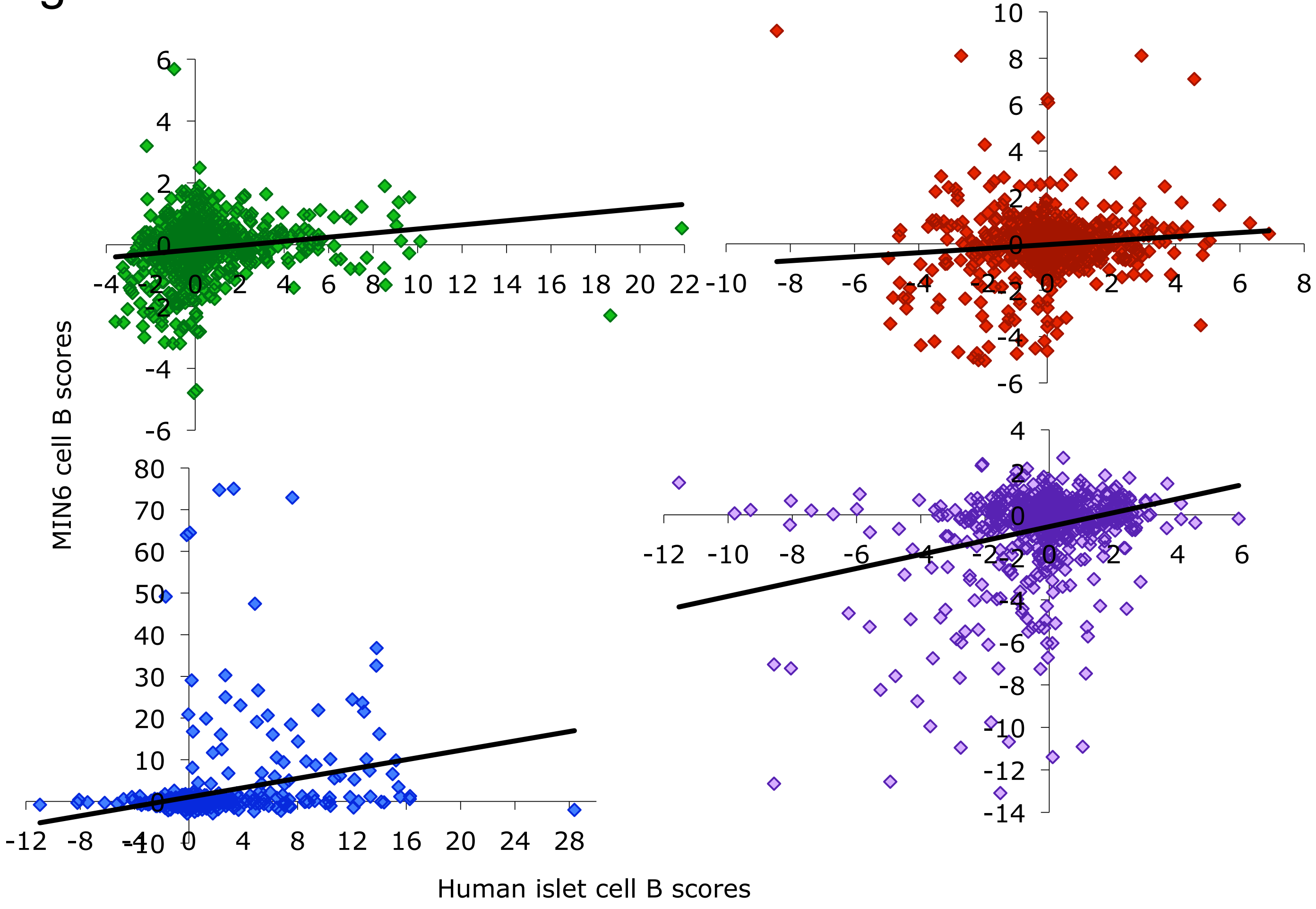

Spearman's rank correlation coefficient of mean MIN6 data and human islet screen.

	S	ρ	p-value
Insulin	44880085	0.2047430	0.000000049
Pdx-1	53095232	0.05917395	0.1186
Hoechst	44375644	0.2136815	0.000000013
Cell number	47665643	0.1553841	0.0000379
